# Investigation and Calculation Method for the Mechanical Properties of Filament Wound Profiles for Deformed Shield Tunnel Reinforcement

**DOI:** 10.3390/ma16041645

**Published:** 2023-02-16

**Authors:** Lei Zhang, Xian Liu

**Affiliations:** College of Civil Engineering, Tongji University, Shanghai 200092, China

**Keywords:** shield tunnel reinforcement, filament wound profiles, mechanical behavior, sensitivity analysis, calculation method

## Abstract

A new type of structural material has begun to be used in the reinforcement of deformed shield tunnels, known as filament wound profiles (FWPs). The FWPs are formed by wrapping carbon-fiber-reinforced polymer (CFRP) around steel tubes that are grouted with concrete inside. However, for practical engineering applications, the design of FWPs requires further insight into their mechanical behavior, since there is no standard method for this at present. In this study, compression and bending tests were carried out to investigate the mechanical behavior of FWPs. A reliable numerical model was established based on the test results, and the effects of the design parameters on the mechanical properties of the FWPs were analyzed qualitatively. The key design parameters of bearing capacity and stiffness were determined through numerical experiments. Based on the experimental results, a method for the calculation of bearing capacities and stiffness was proposed. It was verified that the results of the calculation formulae and the experimental results showed good agreement. Moreover, the results of the formulae were relatively conservative, and most of the errors were within 15%. Thus, this calculation method can be used to calculate the load-bearing capacity and stiffness of FWPs in practical projects.

## 1. Introduction

**Figure 1:**
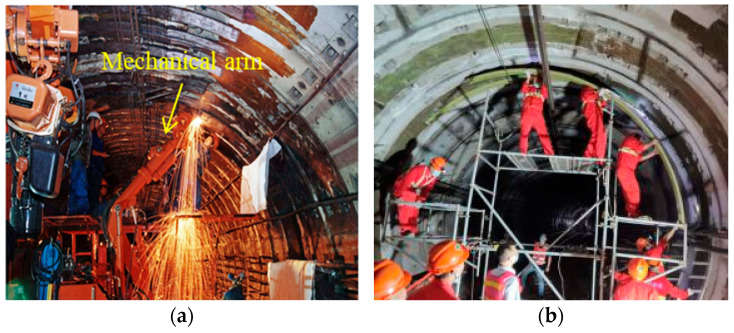
The installation construction in the shield tunnel: (**a**) steel plate; (**b**) filament wound profiles.

The incidence of diseases of shield tunnel linings is gradually increasing with the dual effect of the natural environment and the unexpected changes in loading conditions. Therefore, the demand for shield tunnel reinforcement is also increasing [1]. The methods of reinforcing shield tunnel linings with large deformations include FRP reinforcement [2,3], steel plate reinforcement [4,5,6], steel plate–UHPC composite reinforcement [7,8], and corrugated steel reinforcement [9], among which steel plate reinforcement is widely used in urban metro shield tunnel linings. However, as shown in Table 1, due to the large self-weight of steel plates, mechanical arm support is an indispensable part of the construction, as shown in Figure 1a. With limited time for repair construction in operational tunnels, it is impossible to repair all deformed rings of one tunnel rapidly, because the mechanical arms are scarce and expensive. Therefore, the efficiency of emergency repair of the deformed linings of operational shield tunnels is limited, and a lightweight reinforcement material is urgently needed to improve the efficiency of tunnel repair.

**Figure 2:**
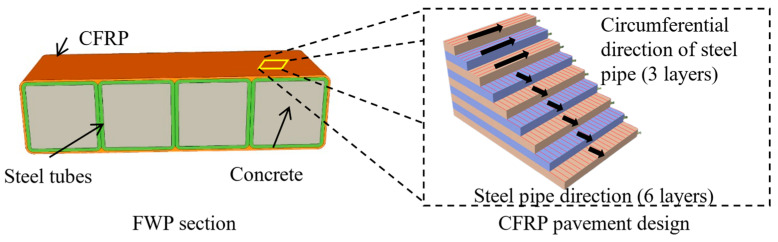
The section of the filament wound profiles and the pavement design of CFRP.

Based on the idea of lightweight structural materials, FWPs have been introduced and applied to reinforce deformed shield tunnel structures in recent years [10]. As shown in Figure 2, the filament wound profiles (FWPs) are formed by wrapping carbon-fiber-reinforced polymer (CFRP) around four steel tubes that are grouted with concrete inside. Because of its light weight, as shown in Table 1 and Figure 1b, the reinforcement construction with non-grouted FWPs could be finished with manual work alone. After installation, the emergency repair of all of the deformed rings in one shield tunnel could be finished simultaneously after grouting in the cavity of the FWPs. With the application of FWPs, the emergency repair efficiency of the reinforcement construction can be greatly improved. Investigation has verified that the effect of FWP reinforcement of deformed shield tunnels is equivalent to that of steel plate reinforcement [10].

As a new kind of structural reinforcement material for shield tunnels, the inspiration of FWPs comes from the concrete-filled CFRP–steel tube (CFRP–CFST) column members that are used in building structures. The mechanical behavior and failure mode of CFRP–CFST under compression [11], bending [12], compression–bending [13], shear [14], and torsion [15] conditions have been investigated, and the compression and bending load-bearing capacity of CFRP–CFST have also been calculated. Similar materials include CFRP-wrapped aluminum tubes [16,17]. However, there are many differences between FWPs used in shield tunnels and CFRP–CFST used in building structures. For example, FWPs are multi-cavity members, and their section size is much smaller than that of CFRP–CFST, while the thickness of CFRP in FWPs is same as the thickness of steel. Therefore, the method of calculating the mechanical properties of CFRP–CFST cannot be directly applied to the FWPs, not to mention the fact that there has been little research conducted on the mechanical behavior of FWPs. A previous investigation concluded that when FWPs were installed at the intrados of the segments, they bore the external loads together with the tunnel linings, and the FWPs were under combined axial force and bending moment [18]. In order to provide a basis for the design of reinforcement for the deformed linings, it is important to investigate the mechanical behavior and the calculation method of the FWPs under axial force and bending moment.

In this study, the mechanical behaviors of FWPs were investigated by component tests, to provide a foundation for the derivation of a method for calculating the mechanical properties of FWPs. Numerous numerical tests were carried out to provide data for the verification of the calculation method. With the proposed new calculation method, the mechanical properties of FWPs can be quantitatively analyzed. Moreover, the design of FWPs can be adjusted flexibly according to the reinforcement requirement of the deformed shield tunnel linings, making the usage of reinforcement materials more economical and efficient.

The rest of this paper is organized as follows: The component tests of the FWPs are carried out, and the mechanical properties of FWPs are investigated and summarized. The numerical tests are carried out to analyze the influence of different design parameters on the mechanical properties of FWPs. Based on the experimental results and the numerical simulation results, the formulae are derived for calculating the ultimate load-bearing capacity and stiffness of FWPs under compression, tension, bending moment, and combined axial force and bending moment. The reliability of the formulae is verified by experimental data.

## 2. Experimental Investigation of the Mechanical Behavior of FWPs

### 2.1. Experimental Program

#### 2.1.1. Specimens

**Figure 3:**
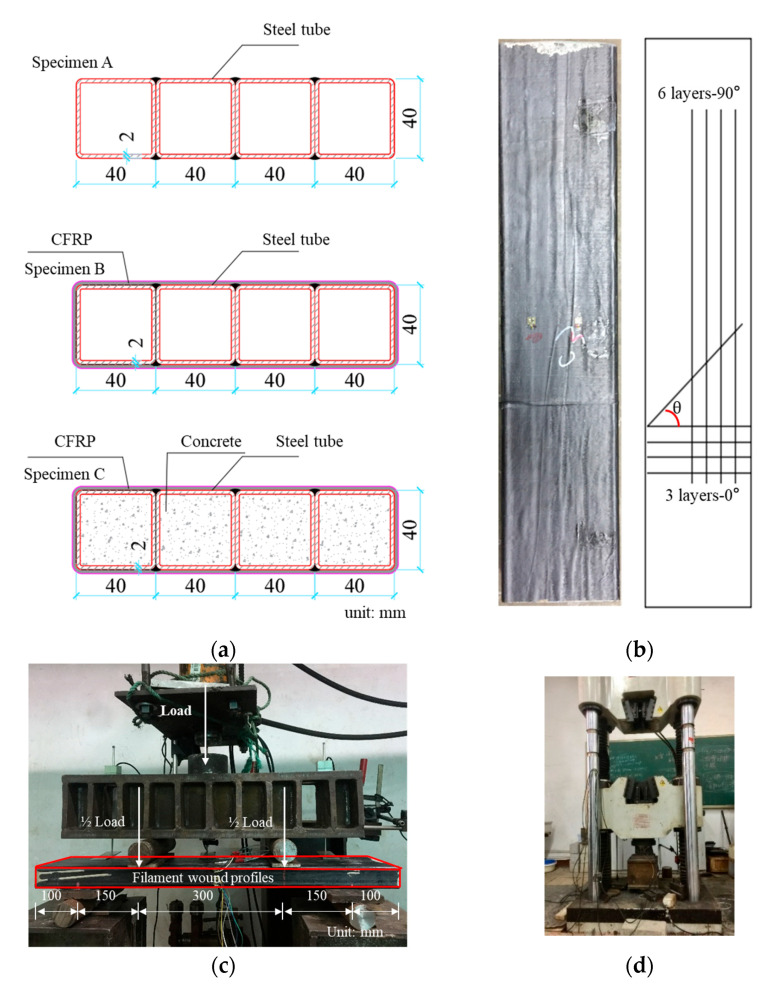
(**a**) Cross-section of a specimen. (**b**) The layup of CFRP. (**c**) Setup of the four-point bending test. (**d**) Compression test.

Three groups of specimens were tested. They referred to intermediate products during the production of FWPs. The latter were produced as follows: Four steel tubes were integrated to a profile (see Figure 3a) by means of welding. The integrated profile refers to specimens of group A. Then, the surface of the integrated steel tubes was subjected to rust removal and sandblasting. Afterwards, they were wrapped in CFRP layers by means of epoxy, resulting in non-grouted FWPs. They were then cured under high pressure for 5 h. The non-grouted FWPs refer to specimens of group B (see Figure 3a). Finally, the cavities of the FWPs were grouted with concrete. The grouted FWPs refer to specimens of group C.

The tubes of the FWPs were composed of Q420 steel. The tensile strength of the CFRP was 4 GPa, and the elastic modulus was 235 GPa. As shown in Figure 3b, the FWPs had 3 layers of CFRP wrapped horizontally and 6 layers of CFRP wrapped vertically. The CFRP wrapped horizontally was marked as 0°, and the CFRP wrapped vertically was marked as 90°. The thickness of each layer was 0.167 mm, and the total thickness of the CFRP was 1.503 mm. The concrete was high-performance self-leveling concrete with a strength of 50 MPa.

#### 2.1.2. Loading Facility and Loading Program

Compression tests of the FWPs were carried out using a static loading method, and the load was applied by a 200 t universal testing machine, as shown in Figure 3d.

Four-point bending tests were carried out in a force-controlled fashion. The tested specimen beam was simply supported (see Figure 3c). The jack force P was imposed on the specimen via a distribution beam. The jack force was increased by 0.05 kN/s up to the load-bearing capacity of the specimens.

#### 2.1.3. Measurement Program

**Figure 4:**
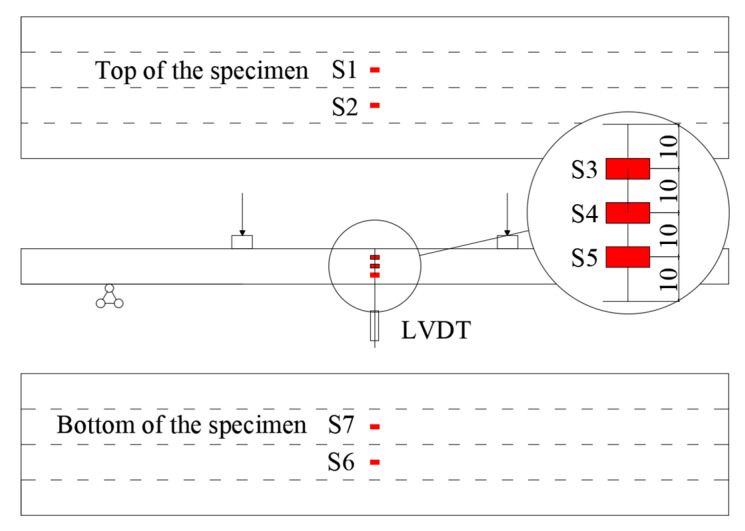
Layout of the points for the monitoring of strain (S1, S2, S3, S4, S5, S6, S7) and deflection (LVDT).

In the bending tests, the experimental measurements concerned the strain and the deflection of the specimens. As for measuring the strains, two gauges were located each on the top and the bottom surface of the specimens. Three gauges were evenly located on the side surface, as shown in Figure 4.

### 2.2. Experiment Results

**Figure 5:**
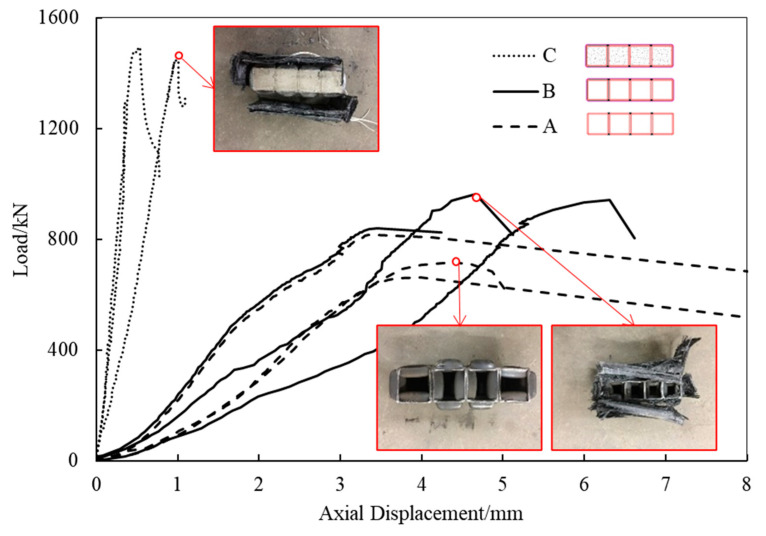
Load–displacement curves of the compression tests.

The compression test results and load–displacement curves of the members are shown in Figure 5 and Table 1. The deformation of group A increased linearly with the increase in the load. When the ultimate load was reached, the steel tubes buckled locally at the load points and lost their stability. The deformation of the non-grouted FWPs increased linearly with the increase in load. When the ultimate load was reached, the adhesive layer between the steel tubes and the CFRP was damaged, and the compressive stiffness of the specimens began to decrease. When the ultimate load-bearing capacity was reached, the steel tubes buckled locally and lost their stability. The wrapped CFRP could not restrict the local buckling of the steel tubes. The deformation of the FWPs increased linearly with the increase in load. When the load reached 1400 kN, the specimen made a lot of “hissing” noise, and the compressive stiffness of the specimen began to decrease. When the ultimate load was reached, the steel tubes buckled locally and the wrapped CFRP could not limit the transverse deformation of the steel tubes, leading to the CFRP tearing at the corners.

**Figure 6:**
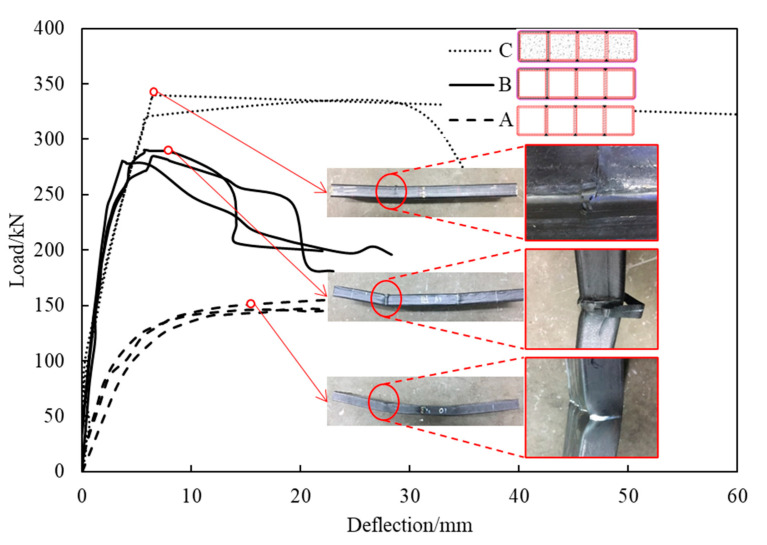
Load–displacement curves of the bending tests.

In the bending tests, there was no visible failure phenomenon of the specimens in group A until the integrated steel tubes yielded. As the displacement increased sharply, associated with the load bucking of the steel tubes at the position of the point loads (see Figure 6), the specific bearing capacity was reached.

The failure mode of the specimens of groups B and C was virtually the same. Their failure process started with the damage to the bonds between the individual CFRP layers, resulting in a hissing sound. Immediately afterwards, the displacement of the tested specimens increased sharply. The failure process ended with tearing of the CFRP at the load points. This phenomenon is of great relevance in engineering practice, because it is a sign of the failure of both non-grouted FWPs and grouted FWPs. The ultimate load-bearing capacity and the stiffness under the bending test are shown in Table 2.

### 2.3. Discussion of the Mechanical Behavior of FWPs

#### 2.3.1. The Function of CFRP in FWPs

The CFRP material has one-way load-bearing characteristics, and it can only bear tensile stress, which means that the six layers of 90° CFRP have no usage in compression tests.

According to the comparison between the compression test results of group A and group B in Table 2, after the steel tubes were restrained by three layers of 0° CFRP, the compressive ultimate load-bearing capacity of the specimen increased by 24.33%. It can also be concluded that the compression stiffness of the FWPs could not be improved by three layers of 0° CFRP. From the compression tests, it could be concluded that 0° CFRP could increase the load-bearing capacity of FWPs but had little relevance to the compressive stiffness.

In the bending tests, the ultimate load-bearing capacity of specimens of group B and group C was 93.0% and 127.2% higher than that of the specimens of group A, respectively (see Table 3 and Figure 6). Both the CFRP and the concrete significantly improved the load-bearing capacity of the FWPs. The bending stiffness was greatly improved by the CFRP according to the slope of the load–displacement curves of group A and B, while it had little relevance to the concrete.

#### 2.3.2. Mechanical Behaviors and Failure Mode

The load was mainly borne by the steel tubes and concrete under compression, with the restriction in transverse deformation due to the CFRP. When the ultimate compressive load-bearing capacity was reached, the concrete underwent transverse deformation, and the steel tubes lost their stability and deformed outwards. The failure mode of the FWPs under compression was that the transverse deformation of the FWPs could not be limited by the CFRP, and the CFRP was torn down.

In the bending tests, six layers of 90° CFRP and steel tubes contributed to the tensile stress together in the tensile area of the section, while the concrete and steel tubes bore the compressive stress together in the compression area of the section. Three layers of 0° CFRP restricted the transverse deformation of the steel tubes in the compression area of the section. When the ultimate bending moment was reached, the 0° CFRP in the compression zone at the loading point could not restrain the transverse deformation of the steel tubes and was torn down, and then the FWPs failed in the bending test. The failure mode of the FWPs under bending moment was that the CFRP was torn down in the compression zone of the FWP section because of the buckling deformation.

## 3. Numerical Tests of the Mechanical Properties of FWPs

### 3.1. Finite Element Modeling and Validation

**Figure 7:**
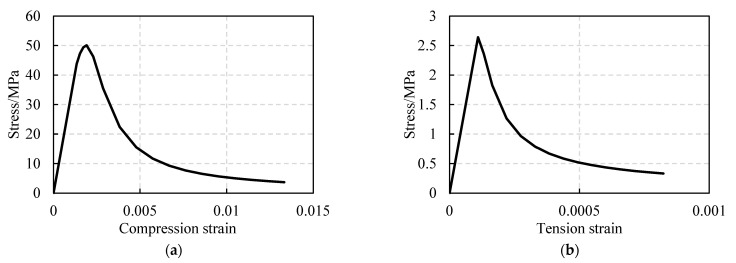
(**a**) Constitutive model of elastoplastic damage for concrete under compression. (**b**) Constitutive model of elastoplastic damage for concrete under tension.

The commercial finite element software ABAQUS6.13/Explicit was employed to simulate FWPs. The material properties are shown in Table 4, Table 5, Table 6 and Table 7. The steel tubes were set as isotropic materials, modeled by the solid elements (C3D8R). The elastic and plastic damage characteristics of CFRP were considered, and the CFRP was discretized by shell elements (S4R). The Hashin damage criterion was considered for plastic damage. After the CFRP material was damaged, the stiffness of the CFRP degraded until it equaled 0, corresponding to the failure mode in the component tests. The stiffness of 0° CFRP degrading to zero means that the CFRP is torn down under tension. The parameter setting of the Hashin damage criterion was derived from research [19,20]. The concrete was modeled by the solid elements (C3D8R). The elastic and plastic damage characteristics of concrete were considered, and the constitutive curves of plastic damage are shown in Figure 7.

The interactions between steel tubes and concrete cubes were simulated by the surface-to-surface contact, and the steel tubes are bound with CFRP shells.

**Figure 8:**
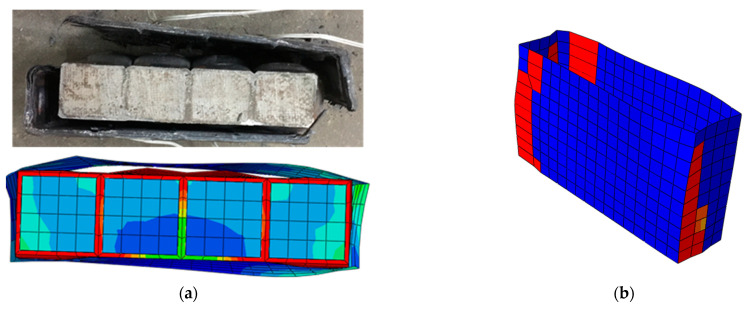

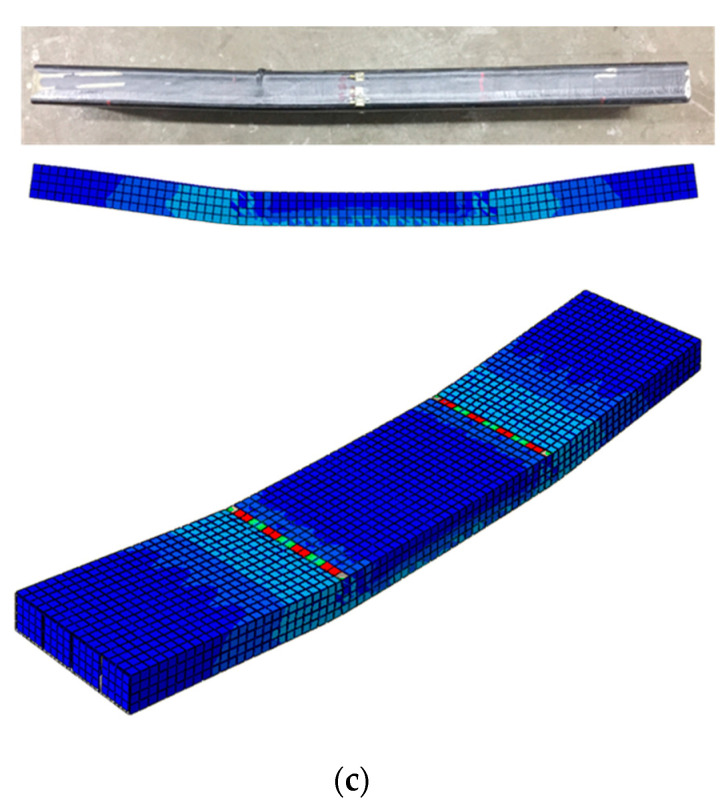
(**a**) FEA model of the compression test. (**b**) The damage to CFRP marked in red. (**c**) FEM model of the bending test.

It can be seen from Figure 8 that the failure mode of FWPs in the numerical model was the same as that in the component tests. The CFRP was torn down at the corners of the FWPs. The position marked in red in the Figure 8b indicates that the stiffness of the CFRP has degraded to 0, showing that the CFRP is torn down at the corner. It can be concluded from Table 8 that the numerical results of FEA are consistent with the test results.

### 3.2. Numerical Test Results of Design Parameters

As shown in Table 9, based on the reliable FEA model, the ultimate load-bearing capacity and stiffness of FWPs under the conditions of tension, compression, and bending were investigated with different design parameters, including concrete grade, steel grade, 0° and 90° layers of CFRP, elastic modulus and strength of CFRP, wrapping angle of CFRP, steel tube thickness, and section size.

**Figure 9:**
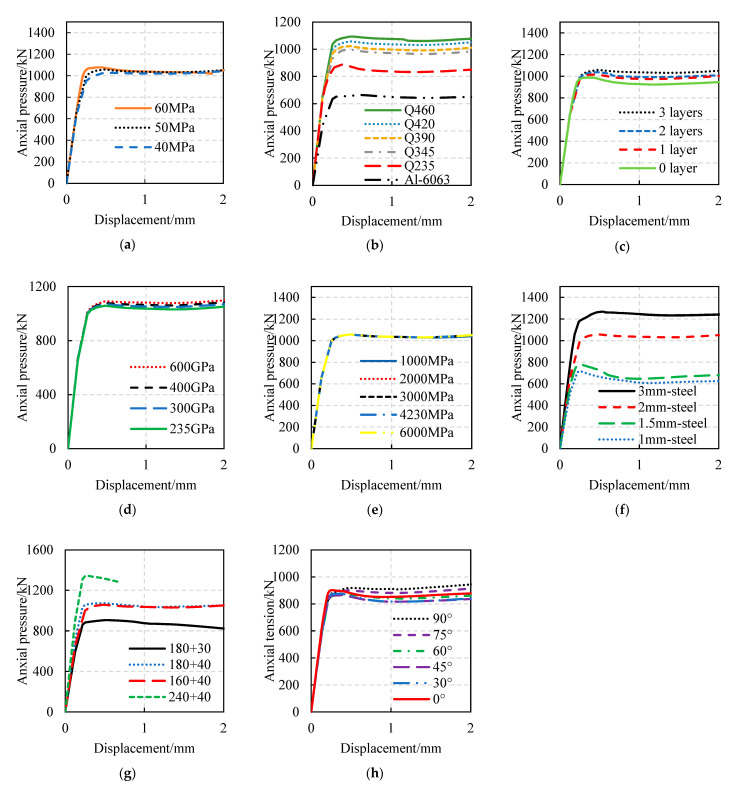
Parameter analysis of compression tests: (**a**) Variable is concrete strength. (**b**) Variable is steel grade. (**c**) Variable is the number of 0° CFRP layers. (**d**) Variable is the elastic modulus of CFRP. (**e**) Variable is the ultimate tensile strength of CFRP. (**f**) Variable is the thickness of the steel tubes. (**g**) Variable is the section size of the FWP. (**h**) Variable is the angle of the CFRP.

**Figure 10:**
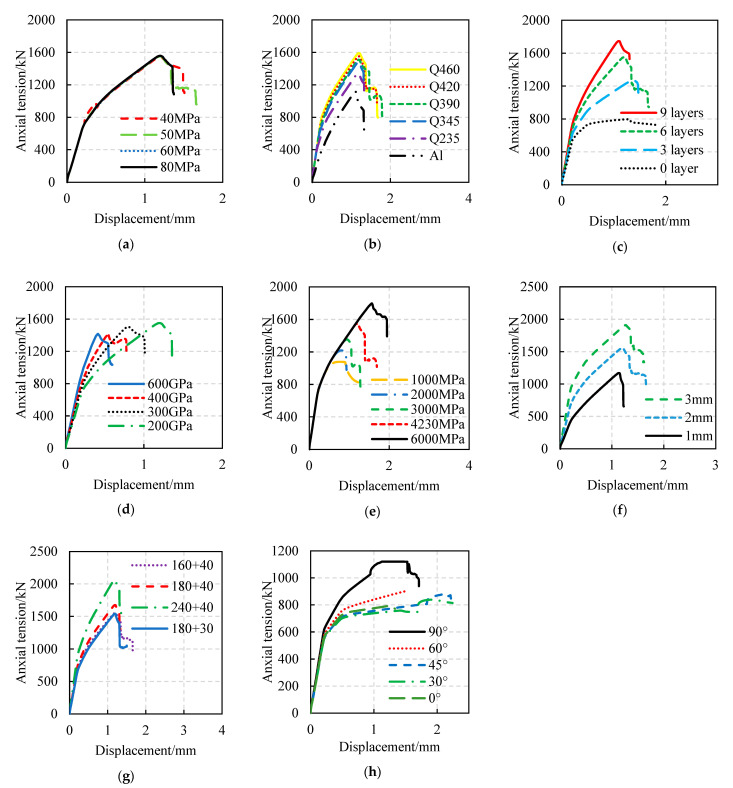
Parameter analysis of tension tests: (**a**) Variable is concrete strength. (**b**) Variable is steel grade. (**c**) Variable is the number of 90° CFRP layers. (**d**) Variable is the elastic modulus of CFRP. (**e**) Variable is the ultimate tensile strength of CFRP. (**f**) Variable is the thickness of the steel tubes. (**g**) Variable is the section size of the FWP. (**h**) Variable is the angle of the CFRP.

**Figure 11:**
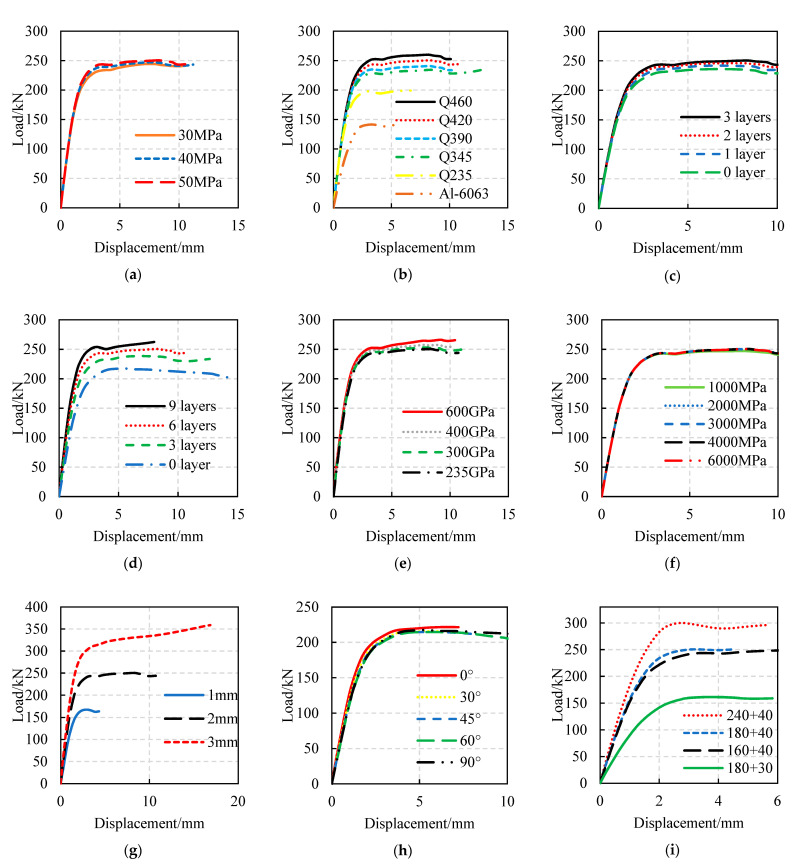
Parameter analysis of bending tests: (**a**) Variable is concrete strength. (**b**) Variable is steel grade. (**c**) Variable is the number of 0° CFRP layers. (**d**) Variable is the number of 90° CFRP layers. (**e**) Variable is the elastic modulus of CFRP. (**f**) Variable is the ultimate tensile strength of CFRP. (**g**) Variable is the thickness of the steel tubes. (**h**) Variable is the angle of the CFRP. (**i**) Variable is the section size of the FWP.

The load–displacement curves of FWPs with different design parameters under axial compression, axial tension, and bending are shown in Figure 9, Figure 10 and Figure 11, respectively. The effects of the design parameters on the mechanical properties of the FWPs are shown in Table 10, where “+” means that the variable is positively correlated with the mechanical properties, “−” means that the variable is negatively correlated with the mechanical properties, and “0” means that the variable is irrelevant to the mechanical properties. When the section size is variable, the order of load-bearing capacity performance of the composite profile members, from small to large, is as follows: 180 × 30, 160 × 40, 180 × 40, 240 × 40.

### 3.3. Sensitivity Analysis

In order to eliminate the influence of the dimensions on the results, the relative value index was applied for analysis. The design parameters and performance indicators were normalized and made dimensionless according to the following formula:

(1) $$R_{p}=\frac{\mathrm{Pu}}{\mathrm{Pur}}$$

(2) $$R_{k}=\frac{\mathrm{Pk}}{\mathrm{Pkr}}$$

(3) $$Z_{i}^{m}=\frac{x_{i}^{m}-\min\left(x^{m}\right)}{\max\left(x^{m}\right)-\max\left(x^{m}\right)}$$

where $R_{p}$ denotes the index of load-bearing capacity, Pu denotes the ultimate load-bearing capacity of the experimental group, Pur stands for the ultimate load-bearing capacity of the benchmark, $R_{k}$ denotes the index of stiffness, Pk denotes the elastic stiffness of the experimental group, Pkr denotes the elastic stiffness of the benchmark, $x^{m}$ denotes the set of parameters m, and $Z_{i}^{m}$ denotes the ith parameter value of the parameter m after normalization.

**Figure 12:**
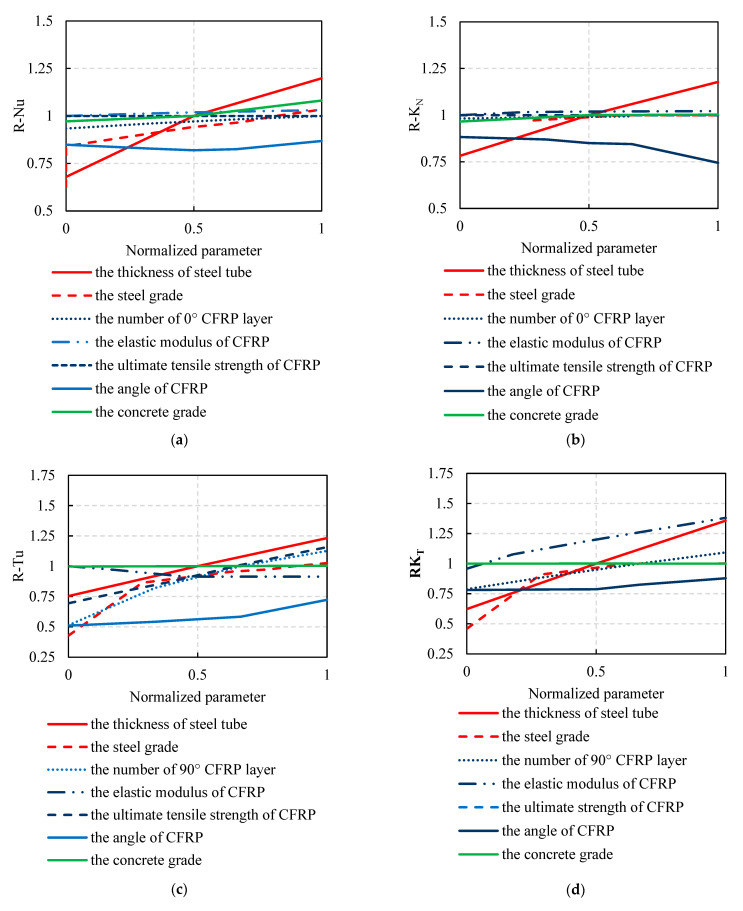

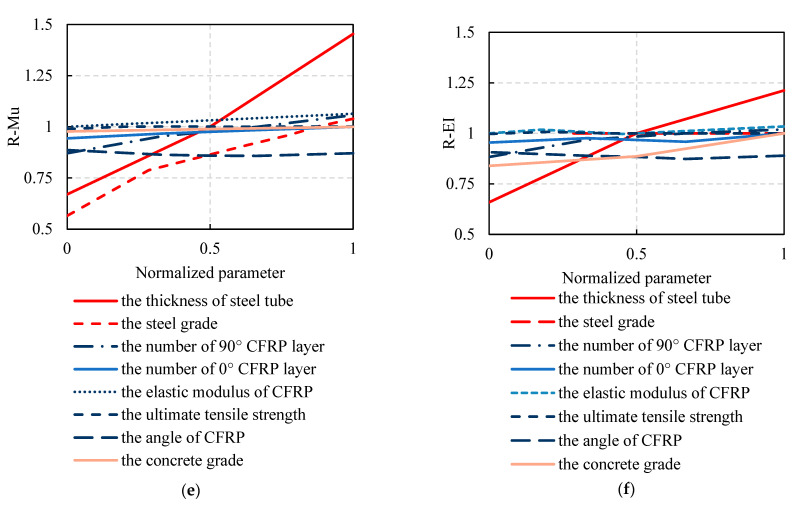
Sensitivity analysis of different design parameters: (**a**) Compressive load-bearing capacity. (**b**) Elastic compressive stiffness. (**c**) Tensile load-bearing capacity. (**d**) Elastic tensile stiffness. (**e**) Ultimate flexural capacity. (**f**) Elastic bending stiffness.

Figure 12 shows the trend of the mechanical properties of FWPs with different design parameters under compression, tension, and bending after normalization.

From Figure 12a, it can be concluded that the ultimate compressive load-bearing capacity ($N_{u}$) of FWPs is mainly affected by three parameters, i.e., steel thickness, steel grade, and the number of 0° CFRP layers. Increasing the thickness of the steel tubes can significantly increase the area of the steel, and increasing the steel grade can increase the ultimate strength of the steel. With more material and a higher level of ultimate compressive stress, the $N_{u}$ of the FWPs improves. The 0° CFRP layers do not directly participate in compression, but they effectively restrict the transverse deformation of concrete-filled steel tubes, delaying the time of buckling of the steel tubes, thereby effectively improving the $N_{u}$ of the FWPs.

From Figure 12b, it can be concluded that the elastic compressive stiffness ($K_{N}$) of FWPs is mainly influenced by the thickness of the steel and the grade of the concrete. The thickness of steel tubes is related to the area of the steel. When the area of the steel increases, the average elastic modulus of the FWPs increases, and the $K_{N}$ increases. The elastic modulus of concrete increases with the increase in the concrete grade, leading to higher $K_{N}$ of the FWPs.

From Figure 12c, it can be concluded that the ultimate tensile load-bearing capacity ($T_{u}$) of FWPs is mainly affected by the steel thickness, the number of 90° CFRP layers, the ultimate tensile strength of the CFRP, and the angle of the CFRP layers. Increasing the thickness of the steel and the number of 90° CFRP layers causes an increase in the area of the tensile materials in the FWP section. With more material participating in the tension, the $T_{u}$ increases. The higher the strength of the CFRP, and the higher the tensile capacity of materials with the same area, the higher the $T_{u}$ of the FWPs. The smaller the angle of the CFRP layers, the smaller the area of CFRP pulled along the steel tube, leading to a decrease in the $T_{u}$ of the FWPs.

From Figure 12d, it can be concluded that the elastic tensile stiffness ($K_{T}$) of FWPs is mainly affected by the steel thickness, the number of 90° CFRP layers, and the elastic modulus of the CFRP. Increasing the steel thickness and the number of 90° CFRP layers ensures that more material participates in tension; therefore, the $K_{N}$ increases. The increase in the elastic modulus of the CFRP ensures that the average elastic modulus of the FWPs is larger and that the $K_{N}$ of the FWPs is increased.

From Figure 12e, it can be concluded that the ultimate bending moment ($M_{u}$) of FWPs is mainly affected by the thickness of the steel, the steel grade, and the number of 90° CFRP layers. The steel area participating in the bending is multiplied with the increase in the thickness of the steel tubes. Increasing the number of 90° CFRP layers ensures that more CFRP can participate in stress at the tension area of the section, enlarging the area of the compression zone in the axially balanced section, and resulting in higher $M_{u}$ of the FWPs.

From Figure 12f, it can be concluded that the bending stiffness (EI) of FWPs is mainly influenced by the thickness of the steel tubes and the number of 90° CFRP layers. Increasing the steel thickness will multiply the average elastic modulus of the material in the compression and tension zones of the section, thereby increasing the EI of the FWPs. The increase in the number of 90° CFRP layers causes more CFRP to participate in the tension at the FWP section, which causes the EI of the FWPs to increase.

## 4. Calculation Method of the Mechanical Properties of FWPs

### 4.1. Bearing Capacity

#### 4.1.1. The Ultimate Compressive and Tensile Load-Bearing Capacity

It can be concluded from Section 2.3.2 that the axial compression load is mainly borne by the steel tubes and concrete in the FWPs. The wrapped CFRP layers restrict the lateral deformation of the concrete-filled steel tubes, improving the ultimate compressive load-bearing capacity ($N_{u}$) of the specimens. Therefore, the $N_{u}$ of the FWPs considers the sum of the load-bearing capacity of the steel tubes and concrete, and the restraint effect coefficient of CFRP ($\xi_{\mathrm{frp}-s}$) should also be considered, as shown in Formulas (4)–(6):

(4) $$N_{u}=A_{s}f_{\mathrm{yy}}+A_{c}f_{c}$$

(5) $$f_{\mathrm{yy}}=(\xi_{\mathrm{frp}-s}+1)f_{y}$$

(6) $$\xi_{\mathrm{frp}-s}=\frac{1}{n}*\frac{A_{\mathrm{frp}\theta }f_{\mathrm{frp}}}{A_{s}f_{y}}$$

where $A_{s}$ and $A_{c}$ are the area of steel and concrete in the FWP section, respectively, $f_{c}$ is the compressive strength of the concrete cubes, $f_{y}$ stands for the yield strength of the steel tubes, $f_{\mathrm{yy}}$ denotes the strength of steel considering 0° CFRP restraint, $\xi_{\mathrm{frp}-s}$ is the correction coefficient of 0° CFRP restraint, $f_{\mathrm{frp}\theta }$ is the ultimate tensile strength of CFRP in the 0° direction, $A_{\mathrm{frp}\theta }$ is the sectional area of the 0° CFRP layers, n is the number of steel tubes in one FWP section, and $f_{\mathrm{frp}}$ is the ultimate tensile strength of the CFRP.

**Figure 13:**
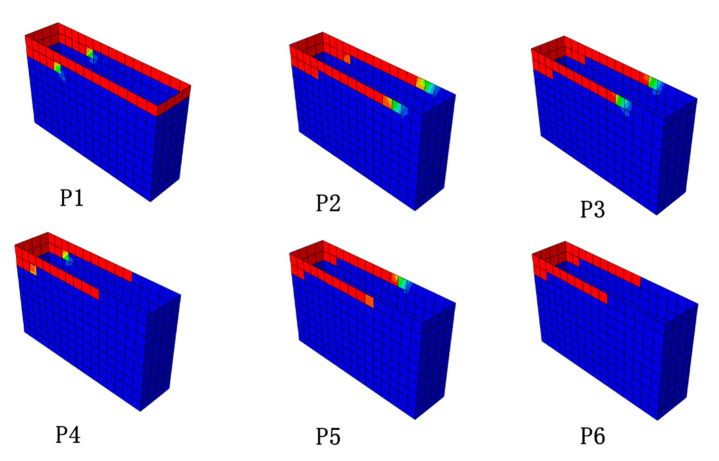
Carbon fiber damage to 6 layers of CFRP under tensile stress conditions (marked with red).

The ultimate tensile load-bearing capacity $\left(N_{\mathrm{ut}}\right)$ refers to the axial tensile load-bearing capacity formula of concrete-filled steel tubular members in the Code [21], and the contribution of 90° CFRP is considered, as shown in Formula (7):

(7) $$N_{\mathrm{ut}}=A_{s}f_{y}+{\mathrm{kA}}_{\mathrm{frp}}f_{\mathrm{frp}}$$

where $A_{\mathrm{frp}}$ denotes the area of 90° CFRP; k stands for the correction coefficient of 90° CFRP strength, and the value of k is 0.503. In the numerical tests, six layers of 90° CFRP in FWPs were damaged after the specimen reached the $N_{\mathrm{ut}}$. As shown in Figure 13, six layers of 90° CFRP did not reach the ultimate strength simultaneously when the FWPs reached $T_{u}$, so the strength of the CFRP needs to be reduced. The solution of the correction coefficient m is based on the numerical test data; a set of contradictory equations was established, and the least-squares method was used to fit it. The value of k was 0.503.

#### 4.1.2. The Ultimate Bending Moment

**Figure 14:**
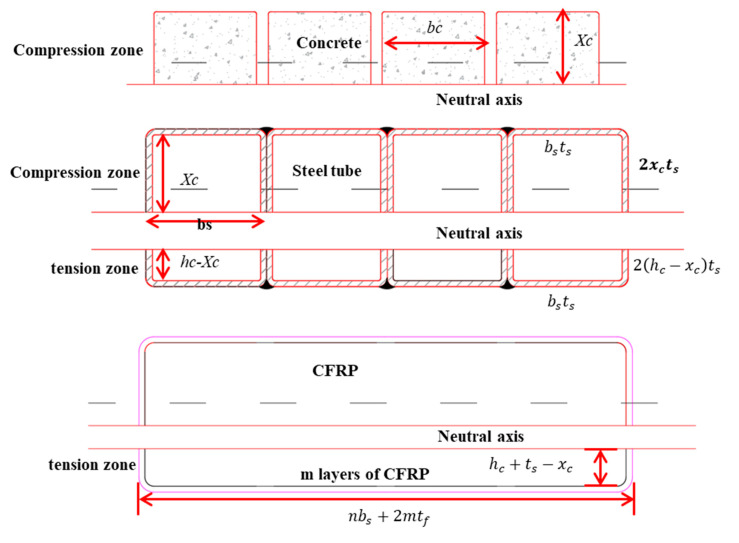
Cross-section of FWP under bending conditions.

As shown in Figure 14, the ultimate bending moment ($M_{u}$) is the superposition of the bending moment values borne by the concrete and the steel tubes in the compression zone, and of the steel tubes and CFRP in the tension zone at the FWP section. During the derivation process, the height of the compression zone of the section was solved by using the boundary condition, with the axial force of the section equal to zero. The stress and moment of each material were calculated according to the assumption of a flat section.

The derivation process adheres to the following basic assumptions:

1. Flat section assumption: The section of FWPs remains flat during bending, without relative slip between materials.
2. Material stress–strain linear elasticity assumption: Concrete compression, steel compression, steel tension, and CFRP tension are all linearly elastic. The contribution of concrete in the tension zone of the section is ignored. The contribution of CFRP to pressure is neglected.
3. The ultimate compressive strain of concrete is 0.0033.
4. Equivalent rectangular stress distribution is used in the stress pattern of concrete in the compressive zone of the section, and the compressive strength is 0.85$f_{c}^{\prime }$

The calculation formula for $M_{u}$ is shown in Formula (8). The bending moment of the concrete ($M_{c}$) is shown in Formula (9). The bending moment of the steel tubes ($M_{s}$) is shown in Formula (10). The bending moment of the CFRP ($M_{\mathrm{cf}}$) is shown in Formula (11).

(8) $$M_{u}=M_{c}+M_{s}+M_{\mathrm{cf}}$$

(9) $$M_{c}=0.85{\mathrm{nf}}_{c}^{\prime }{\beta x}_{c}b_{c}\left(\frac{h_{c}}{2}-\frac{{\beta x}_{c}}{2}\right)$$

(10) $$M_{s}={\mathrm{nt}}_{s}f_{y}\left[x_{c}^{2}+{\left(h_{c}-x_{c}\right)}^{2}+b_{s}\left(h_{c}+t_{s}\right)\right]$$

(11) $$M_{\mathrm{cf}}=\left(b+2{\mathrm{mt}}_{f}\right){\mathrm{mt}}_{f}f_{\mathrm{frp}}\left(h_{c}-x_{c}+t_{s}+\frac{{\mathrm{mt}}_{f}}{2}\right)+2\left(h_{c}-x_{c}+t_{s}\right){\mathrm{mt}}_{f}f_{\mathrm{frp}}\frac{\left(h_{c}-x_{c}+t_{s}\right)}{2}$$

where $f_{c}^{\prime }$ stands for the compressive strength of concrete, β is the rectangular coefficient of equivalent stress under compression, and m is the number of layers of 90° CFRP. The meanings of the other symbols are shown in Figure 14.

#### 4.1.3. FWPs under Compression Bending and Tension Bending Conditions

The FWPs were subjected to compression bending and tension bending in the full-scale test, and the load-bearing capacity calculation formula was derived from the Code [18]. The formula is shown in Formula (12):

(12) $$\frac{N}{N_{u}}+\frac{M}{M_{u}}\leq 1$$

### 4.2. Stiffness

#### 4.2.1. Axial Stiffness

The elastic compressive stiffness ($K_{N}$) of the FWPs is mainly contributed by the concrete and steel tubes. The elastic tensile stiffness ($K_{T}$) is mainly contributed by the CFRP and steel tubes, and the contribution of the concrete can be ignored. Therefore, the calculation formula of tension and compression stiffness can be established, as shown in Formulae (13) and (14):

(13) $$K_{T}=E_{s}A_{s}+E_{\mathrm{frp}}A_{\mathrm{frp}}$$

(14) $$K_{N}=E_{s}A_{s}+E_{c}A_{c}$$

where $E_{s}$, $E_{c}$, and $E_{\mathrm{frp}}$ represent the elastic modulus of steel, concrete, and CFRP, respectively.

#### 4.2.2. Bending Stiffness

Based on the derivation process of Formula (8), the bending stiffness (EI) calculation formula of FWPs is summarized in Formula (15). The moment of inertia provided by the concrete ($I_{c}$) is shown in Formula (16). The moment of inertia provided by the steel tubes ($I_{s}$) is shown in Formula (17). The moment of inertia provided by the CFRP ($I_{\mathrm{frp}}$) is shown in Formula (18). The other symbols’ meanings are as defined above.

(15) $$\mathrm{EI}=E_{\mathrm{frp}}I_{\mathrm{frp}}+E_{s}I_{s}+E_{c}I_{c}$$

(16) $$I_{c}=\frac{n}{3}b_{c}x_{c}^{3}$$

(17) $$I_{s}==\frac{n}{6}b_{s}t_{s}^{3}+{\mathrm{nb}}_{s}t_{s}\left[{\left(x_{c}+\frac{t_{s}}{2}\right)}^{2}+{\left(h_{c}-x_{c}+\frac{t_{s}}{2}\right)}^{2}\right]+2n\left[\frac{1}{3}t_{s}x_{c}^{3}+\frac{1}{3}t_{s}{\left(h_{c}-x_{c}\right)}^{3}\right]$$

(18) $$I_{f}==\frac{2}{3}{\mathrm{mt}}_{f}{\left(h_{c}-x_{c}+t_{s}\right)}^{3}+\left[\frac{1}{12}b_{f}{\left({\mathrm{mt}}_{f}\right)}^{3}+b_{f}{\mathrm{mt}}_{f}{\left(h_{c}-x_{c}+t_{s}+\frac{{\mathrm{mt}}_{f}}{2}\right)}^{2}\right]$$

## 5. Validation and Limitations

### 5.1. Validation of the Load-Bearing Capacity Formula

#### 5.1.1. Single Stress State

**Figure 15:**
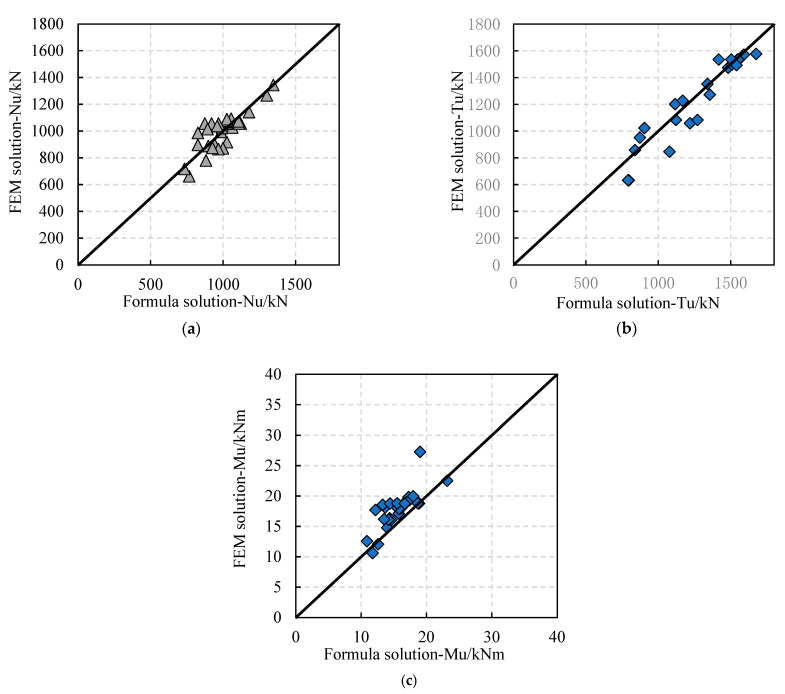
Comparison between formula solutions and test results: (**a**) ultimate compressive load-bearing capacity; (**b**) ultimate tension load-bearing capacity; (**c**) ultimate bending capacity.

As shown in Figure 15, the abscissa of the symbol is the formula solution of Nu, Tu and Mu, and the ordinate of the symbol is the FEM solution of Nu, Tu and Mu. The closer the symbols are to the slash, the closer the two values are. Thus, the validity of the load-bearing capacity formula is verified. The comparison between the calculation formula and the test results is shown in Table 11. The calculation results of the ultimate load-bearing capacity formula are conservative and smaller than the actual test values, and the standard deviation, coefficient of variation, and ratio fluctuation are small, meaning that the load-bearing capacity formula can be referred to in the actual design calculation.

#### 5.1.2. Combined Stress State

In this section, a simplified computational model is established to calculate the N–M correlation curve of FWPs and improve the calculation efficiency of the finite element model.

The simplified computational model keeps the upper-edge strain of the FWP section constant during calculation, and the lower-edge strain of the FWP section changes from compression to tension. In each incremental calculation step, the axial force N and bending moment M of the composite profile are calculated in incremental steps according to Formulas (19) and (20). After summarizing the $\left(M_{t},N_{t}\right)\;$ obtained in each incremental step, the corresponding N–M curve can be drawn.

(19) $$N_{t}=\overset{n}{\underset{i=1}{\sum }}\left(\sigma_{\mathrm{ifrp}}A_{\mathrm{ifrp}}+\sigma_{\mathrm{ic}}A_{\mathrm{ic}}+\sigma_{\mathrm{is}}A_{\mathrm{is}}\right)$$

(20) $$M_{t}=\overset{n}{\underset{i=1}{\sum }}\left(\sigma_{\mathrm{ifrp}}A_{\mathrm{ifrp}}y_{\mathrm{ifrp}}+\sigma_{\mathrm{ic}}A_{\mathrm{ic}}y_{\mathrm{ic}}+\sigma_{\mathrm{is}}A_{\mathrm{is}}y_{\mathrm{is}}\right)$$

where $\sigma_{\mathrm{ifrp}}$, $\sigma_{\mathrm{ic}}$, and $\sigma_{\mathrm{is}}$ denote the stress of CFRP, concrete, and steel in layer I, respectively. $A_{\mathrm{ifrp}}$, $A_{\mathrm{ic}}$, and $A_{\mathrm{is}}$ stand for the area of CFRP, concrete, and steel in layer i, respectively. $y_{\mathrm{ifrp}}$, $y_{\mathrm{ic}}$, and $y_{\mathrm{is}}$ are the distance from the ith layer of CFRP, concrete, and steel to the neutral axis, respectively.

The following assumptions are considered in the calculation process:

1. Plane section assumption of the FWP section;
2. The influence of local buckling of the steel tubes is not considered;
3. The tension of the concrete is ignored;
4. CFRP can only bear the tension in the fiber direction.

**Figure 16:**
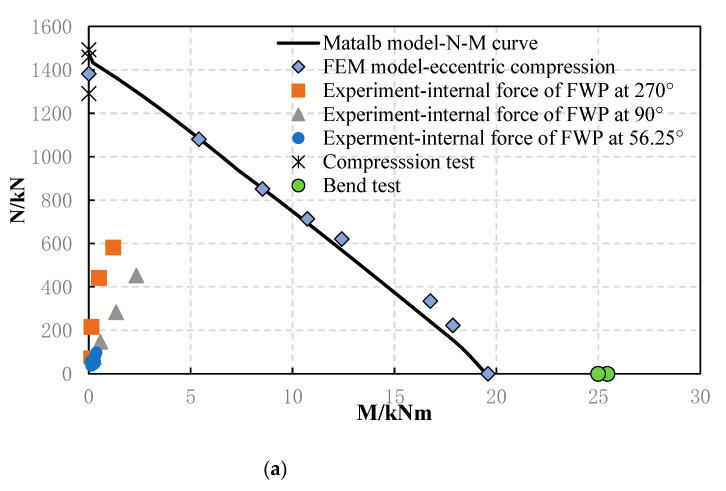

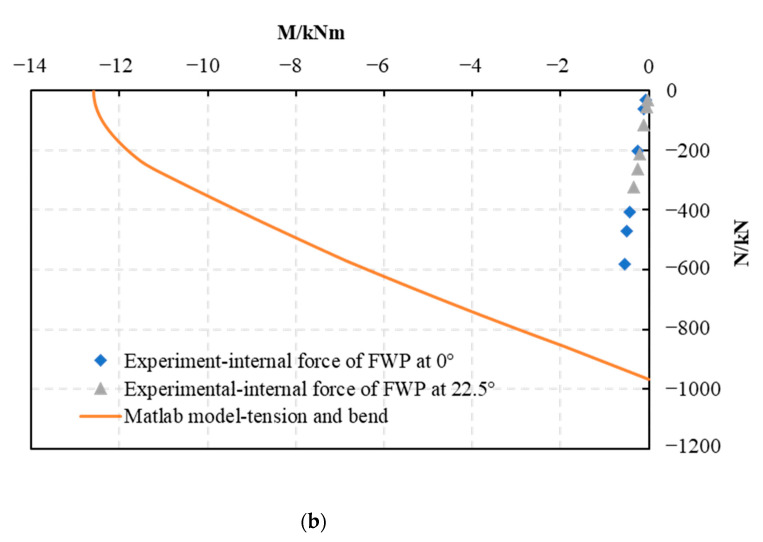
Comparison between the simplified calculation model solutions and the experimental results: (**a**) compression–bending conditions; (**b**) tension–bending conditions.

As shown in Figure 16, the simplified computational model’s results were compared with the results of the component tests and numerical tests. It can be concluded that the component test results, numerical test results, and simplified computational model results fit well. According to the results of the full-scale testing of FWPs used to strengthen staggered-jointed tunnel linings [15], the FWPs in the 56.25°, 90°, and 270° sections were still elastic. Therefore, the simplified computational model can be used for verification.

**Figure 17:**
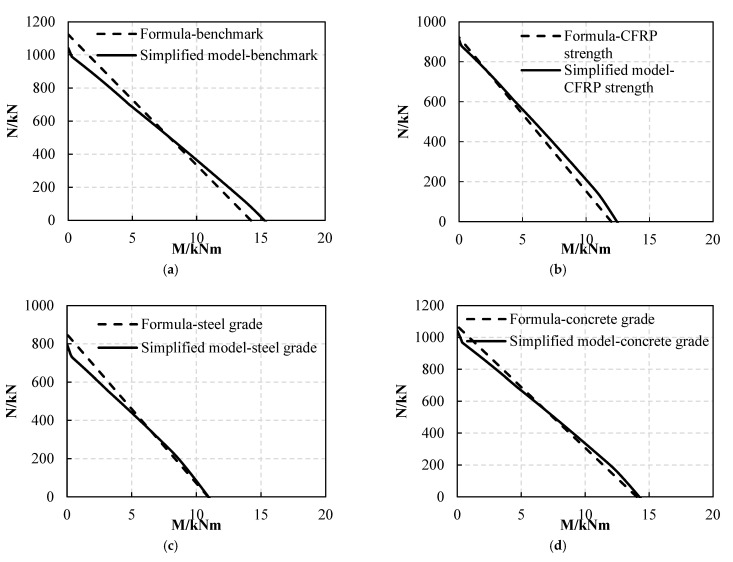

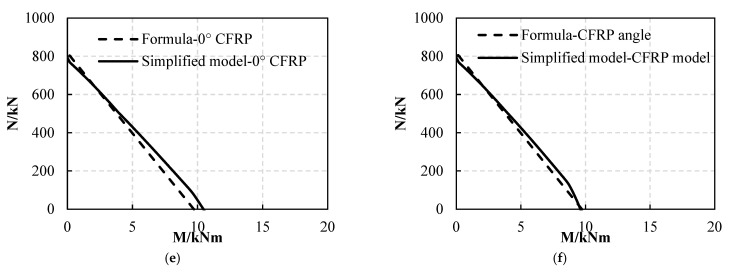
Comparison between formula results and simplified calculation model solutions: (**a**) benchmark; (**b**) variable: CFRP strength is 2000 MPa; (**c**) variable: steel grade is Q235; (**d**) variable: concrete grade is C40; (**e**) variable: the number of 0° CFRP layers is 0; (**f**) variable: the angle of 3 CFRP layers is 90°.

It can be seen from Figure 17 that the load-bearing capacity calculation formula under combined stress states is consistent with the simplified computational model’s results, so Formula (12) can be used to calculate the load-bearing capacity of FWPs under compression bending and tension bending conditions.

### 5.2. Validation of Stiffness Formula

**Figure 18:**
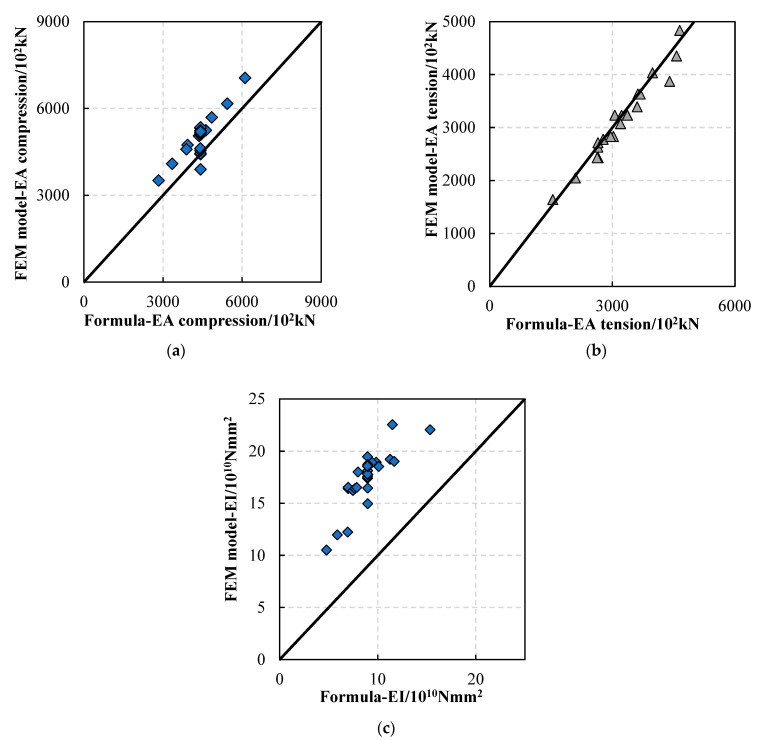
Comparison between the formula results and the numerical model results: (**a**) compression stiffness; (**b**) tension stiffness; (**c**) bending stiffness.

The comparison between the $K_{N}$ and $K_{T}$ formula solutions and the test results is shown in Figure 18a,b below. The average value of the ratio between the $K_{N}$ formula calculation results and the test results was 0.87, the standard deviation was 0.068, and the coefficient of variation was 0.078. The average value of the ratio between the calculated results of the $K_{T}$ formula and the test results was 0.97, the standard deviation was 0.039, and the coefficient of variation was 0.04. The formula calculation results are conservative, meaning that they can be referred to in the actual design calculation.

The comparison between the formula of EI and the numerical model’s calculation results is shown in Figure 18c below. The stiffness calculated by the numerical tests was generally larger than that calculated by the EI formula. The average value of the ratio between the results of the EI formula and the test results was 0.49, the standard deviation was 0.0405, and the coefficient of variation was 0.008, as shown in Table 12. In summary, the formula for calculating the EI of FWPs is conservative and can be used for reference in the actual design calculation.

### 5.3. Limitations

The calculation method proposed here is based on the working mechanism of the FWPs. The formula is simple and can be calculated by hand. However, the results of the calculation method are relatively conservative, and the errors are within 15% according to Table 10 and Table 11, except for the formula of bending stiffness. The calculation method results are less discrete from the test results.

There have been few compression and bending tests carried out to verify the accuracy of the calculation formulae to date, but it could also be verified by new component tests and numerical simulations for the practical application of tunnel reinforcements in the future. New approaches, such as neural networks [22,23], could be used to propose new methods for predicting the mechanical properties of FWPs in the future.

## 6. Conclusions

In this paper, the mechanical behavior of FWPs was clarified through component tests, and a reliable FEA model of FWPs was established. The effects of the design parameters were qualitatively summarized through parameter analysis. Based on the mechanical behaviors of FWPs and the data of FEA tests, the load-bearing capacity and stiffness calculation formula of the FWPs were established and verified. The conclusions are as follows:

1. From the compression test, it can be concluded that the steel tubes and concrete bear the compressive load when the FWPs are compressed, and the 0° CFRP improves the compressive ultimate load-bearing capacity by limiting the transverse deformation of the steel tubes. The compressive failure mode is that three layers of 0° CFRP cannot limit the transverse deformation of the steel tubes, and then the CFRP is torn down.
2. When the FWPs bear the bending moment, the concrete cubes and the steel tubes in the compression area at the FWP section bear the compressive stress, while the steel tubes and the six layers of 90° CFRP in the tension area bear the tensile stress. The bending failure mode is that the steel tubes in the compression area of the section buckle and deform outwards at the loading point, while the 0° CFRP cannot limit the transverse deformation of the steel tubes, and the 0° CFRP is torn down.
3. According to the sensitivity analysis of the load-bearing capacity parameters, the key parameters of compressive ultimate load-bearing capacity of FWPs are steel thickness, steel grade, and the number of 0° CFRP layers. The key parameters of ultimate tensile load-bearing capacity are steel thickness, number of 90° CFRP layers, and CFRP strength; The key sensitive parameters of ultimate bending capacity are steel thickness, steel grade, and number of 90° CFRP layers.
4. According to the sensitivity analysis of the stiffness parameters, the key parameters of compressive stiffness are steel thickness and concrete grade. The key parameters of tensile stiffness are the steel tube thickness, the number of 90° CFRP layers, and the CFRP elastic modulus. The key parameters of bending stiffness are the thickness of the steel tubes and the number of 90° CFRP layers.
5. According to the mechanical behavior of FWPs, the load-bearing capacity and stiffness calculation formulae for FWPs under tension, compression, bending, compression–bending, and tension–bending conditions were derived and established. The formula results are conservative and consistent with the component test results, FEA test results, and theoretical calculation results. Most of the errors are within 15%, which means the calculation method can be used for actual design.

The formula proposed in this paper for calculating the mechanical properties of the FWPs lays the foundation for subsequent research on the design method of FWP-reinforced shield tunnels.

## Figures and Tables

**Table 1:** The comparison of single parts’ self-weight between steel plate reinforcement and FWP reinforcement.

Type	Single Part	Self-Weight
Steel plate reinforcement	60° steel plate with 20 mm thickness [3]	384 kg
FWP reinforcement	100° FWPs [7]	46 kg

**Table 2:** The results of the compression tests.

Specimen	Compression Bearing Capacity/kN	Average Value/kN	Displacement/mm	Average Value/mm
Compression-A1	718.01	732.43	4.16	3.88
Compression-A2	815.16		3.29	
Compression-A3	664.12		4.20	
Compression-B1	822.24	910.66	3.35	4.98
Compression-B2	942.28		6.42	
Compression-B3	967.47		5.17	
Compression-C1	1492.49	1414.59	0.38	0.37
Compression-C2	1291.60		0.36	
Compression-C3	1459.67		1.01	

**Table 3:** The results of the bending tests.

Specimen	Load/kN	Bending Bearing Capacity t/kNm	Average Value/kNm	Displacement/mm	Average Value/mm
Bending-A1	150.03	11.25	11.09	15.73	19.58
Bending-A2	146.42	10.98		20.49	
Bending-A3	147.23	11.04		22.52	
Bending-B1	290.43	21.78	21.41	8.89	9.36
Bending-B2	280.61	21.05		10.09	
Bending-B3	285.21	21.39		9.10	
Bending-C1	339	25.43	25.20	6.44	6.23
Bending-C2	333	24.98		6.02	

**Table 4:** The properties of steel tubes in the numerical model.

	Value	Unit
Density	7.8 × 10^−9^	$t/{\mathrm{mm}}^{3}$
Elastic modulus	200,000	MPa
Poisson’s ratio	0.3	-
Yield stress	420	MPa

**Table 5:** The properties of CFRP in the numerical model.

	Value	Unit
Density	1 × 10^−9^	t/${\mathrm{mm}}^{3}$
Elastic modulus-E1	235,000	MPa
Poisson’s ratio	0.05	-

**Table 6:** Hashin failure criteria parameters.

	Parameter	Value
Damage initiation parameter/MPa	Longitudinal tensile strength	4000
	Longitudinal compressive strength	267
	Transverse tensile strength	643
	Transverse compressive strength	267
	Longitudinal shear strength	643
	Transverse shear strength	267
Fracture energy/(mJ/mm)	Longitudinal tensile fracture energy	223.66
	Longitudinal compressive fracture energy	72
	Transverse tensile fracture energy	83
	Transverse compressive fracture energy	72

**Table 7:** The properties of concrete in the numerical model.

	Value	Unit
Density	2.4 × 10^−9^	t/mm^3^
Elastic modulus	34,500	MPa
Poisson’s ratio	0.2	-

**Table 8:** Comparison between experimental results and FEA results.

	Ultimate Compressive Bearing Capacity/kN	Compressive Stiffness in Elastic Stage/10^2^ kNmm	Ultimate Bending Bearing Capacity/kN	Bending Stiffness in Elastic Stage/10^10^ Nmm^2^
Experiment results	1412	3821	336	12.3
Numerical model results	1382	4896	297	16.7
Experiment/numerical model	97.9%	128.1%	88.4%	135.8%

**Table 9:** Design parameter variables.

	Design Parameter	Variables
1	Concrete grade	C30, C40, C50, C60
2	Steel grade	Q235, Q345, Q390, Q420, Q460, Al-6063-T5
3	The thickness of steel tubes	1 mm, 2 mm, 3 mm
4	The number of 90° CFRP layers	0, 3, 6, 9
5	The number of 0° CFRP layers	0, 1, 2, 3
6	The elastic modulus of CFRP	200 GPa, 300 GPa, 400 GPa, 600 GPa
7	The strength of CFRP	2000 MPa, 3000 MPa, 4000 MPa, 6000 MPa
8	The wrapping angle of CFRP	0°, 30°, 45°, 60°, 90°
9	Section size of FWPs	40 mm × 160 mm, 30 mm × 180 mm, 40 mm × 180 mm, 40 mm × 240 mm

**Table 10:** Summary of effects of design parameters.

	Design Parameter	Nc	Kc	Nt	Kt	Mu	K_EI_
1	Concrete grade	+	+	0	0	+	+
2	Steel grade	+	0	+	0	+	0
3	The thickness of steel tubes	+	+	+	+	+	+
4	The number of 90° CFRP layers	0	0	+	+	+	+
5	The number of 0° CFRP layers	+	0	0	0	+	0
6	The elastic modulus of CFRP	+	+	−	+	+	+
7	The strength of CFRP	0	0	+	0	0	0
8	The wrapping angle of CFRP	<45°− >45°+	0	−	−	<60°− >60°+	<60°− >60°+
9	Section size of FWPs	+	+	0	0	+	+

“+” means positive correlation, “−” means negative correlation, and “0” means irrelevant.

**Table 11:** Reliability verification of the load-bearing capacity formula.

	Formula/Experiment	Standard Deviation	Coefficient of Variation
Compression	0.99	0.085	0.0855
Tension	0.98	0.0948	0.0967
Bending	0.87	0.0816	0.0938

**Table 12:** Reliability verification of the stiffness formula.

	Formula/Experiment	Standard Deviation	Coefficient of Variation
Compression	0.87	0.068	0.078
Tension	0.97	0.039	0.04
Bending	0.49	0.0405	0.008

## Data Availability

Not applicable.
